# Optical Coherent Tomography as a Potential Biomarker for Logopenic Variant Primary Progressive Aphasia: A Cross‐Sectional Prospective Study

**DOI:** 10.1002/alz70856_098816

**Published:** 2025-12-24

**Authors:** Fei Wu, Ali Dirani, Monica Lavoie, Mélanie Hébert, Robert Laforce

**Affiliations:** ^1^ Clinique Interdisciplinaire de Mémoire, CHU de Québec‐Université Laval, Québec, QC, Canada; ^2^ Laval University Medical School, Québec, QC, Canada; ^3^ Centre universitaire d'ophtalmologie ‐ CHU de Québec ‐ Université Laval, Québec, QC, Canada; ^4^ Research Chair on Primary Progressive Aphasia ‐ Fondation de la famille Lemaire, Quebec, QC, Canada; ^5^ Clinique Interdisciplinaire de mémoire, CHU de Québec ‐ Université Laval, Quebec City, QC, Canada

## Abstract

**Background:**

The logopenic variant of Primary Progressive Aphasia (lvPPA) is a neurodegenerative disorder affecting primarily language functions. In 86% of lvPPA cases, the underlying pathology is amyloidopathy, as seen in Alzheimer's disease (AD). Since the retina is considered an extension of the brain, recent research has explored optical coherence tomography (OCT) and OCT‐angiography (OCT‐A) as non‐invasive biomarkers in AD. However, their potential in lvPPA remains unexplored. The aim of this study was to compare retinal findings in lvPPA patients and healthy controls using OCT and OCT‐A.

**Methods:**

We conducted a cross‐sectional study recruiting participants with lvPPA (diagnostic criteria by Gorno‐Tempini, 2011) and healthy controls matched for sex and age. Participants were excluded if they had preexisting neurological or eye conditions. An extensive ophthalmological assessment was conducted to rule out eye diseases. OCT/OCTA imaging was then performed for all participants. For lvPPA patients, the Clinical Dementia Rating scale and a lumbar puncture were performed to assess disease stage and underlying pathology.

**Results:**

Ten lvPPA patients and eleven controls were enrolled. All lvPPA patients had amyloidopathy confirmed by lumbar puncture. Mean CDR global score was 0.55 ± 0.16 (indicating mild dementia). Retinal nerve fiber layer (RNFL) in the temporal region was significantly thinner in the lvPPA group compared to controls (63.1 ± 3.3 μm vs 75.6 ± 3.2 μm, *p* = 0.013). Foveal avascular zone (FAZ) circularity was also significantly lower in the lvPPA group (0.69 ± 0.02 vs 0.77 ± 0.02, *p* = 0.002).

**Conclusions:**

Our findings suggest decreased RNFL thickness and reduced FAZ circularity in lvPPA. Decreased RNFL thickness reflects neuronal degeneration and its underlying mechanisms include retinal amyloid accumulation or retrograde degeneration. This suggests that amyloid‐induced brain atrophy leads to a lack of trophic factors, resulting in thinning of the RNFL while reduced FAZ circularity could signal early vascular alterations induced by amyloid. Altogether, these findings suggest that OCT and OCT‐A could serve as valuable biomarkers for lvPPA, thus enhancing early diagnosis.

